# Point cloud registration method for maize plants based on conical surface fitting—ICP

**DOI:** 10.1038/s41598-022-10921-6

**Published:** 2022-04-27

**Authors:** Kai’xing Zhang, He Chen, Hao Wu, Xiu’yan Zhao, Chang’an Zhou

**Affiliations:** 1 College of Mechanical and Electronic Engineering, Shandong Agricultural University, Taian, 271018 China; 2 College of Information Science and Engineering, Shandong Agricultural University, Taian, 271018 China

**Keywords:** Civil engineering, Engineering

## Abstract

Reconstructing three-dimensional (3D) point cloud model of maize plants can provide reliable data for its growth observation and agricultural machinery research. The existing data collection systems and registration methods have low collection efficiency and poor registration accuracy. A point cloud registration method for maize plants based on conical surface fitting—iterative closest point (ICP) with automatic point cloud collection platform was proposed in this paper. Firstly, a Kinect V2 was selected to cooperate with an automatic point cloud collection platform to collect multi-angle point clouds. Then, the conical surface fitting algorithm was employed to fit the point clouds of the flowerpot wall to acquire the fitted rotation axis for coarse registration. Finally, the interval ICP registration algorithm was used for precise registration, and the Delaunay triangle meshing algorithm was chosen to triangulate the point clouds of maize plants. The maize plant at the flowering and kernel stage was selected for reconstruction experiments, the results show that: the full-angle registration takes 57.32 s, and the registration mean distance error is 1.98 mm. The measured value’s relative errors between the reconstructed model and the material object of maize plant are controlled within 5%, the reconstructed model can replace maize plants for research.

## Introduction

With the rapid development of computer vision technology, the research on 3D point cloud models is gradually becoming a hotspot in agricultural 3D reconstruction. Using 3D reconstruction technology to build the 3D point cloud reconstruction model of maize plants can provide reliable data for growth observation and agricultural machinery research of maize plants, saving human and material resources and improving the efficiency and reliability of research. However, there were few studies on 3D reconstruction of maize plants. Due to the difference from industrial products with regular shapes, the irregular surface of the naturally grown maize plants makes it difficult to collect and register the point clouds. Therefore, improving the collection accuracy of raw data and the registration accuracy have become the key of the maize plants’ reconstruction research. Data Collection and registration are essential steps in the 3D reconstruction process. The data collection plays a fundamental role. And the choice of registration method determines the registration accuracy directly. However, many currently published data collection systems and registration methods cannot meet the requirements of high-precision 3D reconstruction.

The data collection system provides the basic raw data; a number of different data collection systems have been established for the 3D reconstruction of plants in recent years. Medeiros et al.^1^ used a lidar to obtain 3D point clouds of fruit trees for reconstruction. Yang et al.^2^ established an image collection platform with a BB2-08S2C-60 binocular camera to complete the identification and reconstruction of citrus fruit branches. Liang et al.^3^ exploited visual structure from motion (SfM) software based on the SfM algorithm to collect point clouds of maize plants from different perspectives for 3D reconstruction. Pierzchała et al.^4^ built a system with a lidar and a stereo camera to construct a 3D map of forest areas. Botterill et al.^5^ chose a Red–Green–Blue (RGB) camera to collect the data of grape branches and used the SfM algorithm for 3D reconstruction. Karkee et al.^6^ employed an RGB camera and a depth camera based on the time-of-flight (TOF) principle to build a system to reconstruct part of the apple trees, but the 3D images were taken from only one side of each tree in this research. Although the above data collection systems can complete the collection task, lots of problems still exist, such as unsatisfactory data collection, complex collection process, and long collection time in the collection process, etc., which lead to low collection efficiency and inadequate registration accuracy.

Kinect V2 is the second-generation depth camera developed and manufactured by Microsoft Corporation. With its low price and excellent data collection capabilities, it has been widely used in the 3D reconstruction. For instance, Chattopadhyay et al.^7^ utilized a Kinect V2 camera to reconstruct 3D models of the main trunks and main branches of apple trees, the reconstruction error of the main branches could be guaranteed within 5 mm. Akbar et al.^8^ proposed a 3D reconstruction method based on semicircle fitting and used a Kinect V2 camera to obtain depth images of apple trees for reconstruction. Zhou et al.^9^ used a Kinect V2 camera to collect point clouds from four different viewpoints and proposed a point cloud registration method based on calibration spheres to reconstruct trees. The above studies obtained fine results by employing Kinect V2 to collect point clouds, but there is still an inconvenience that point clouds from multiple angles cannot be automatically collected.

The registration process fuses the collected data into a complete object; many registration methods have emerged to reconstruct plants. Li et al.^10^ used the adaptive support weight (ASW) algorithm to establish a binocular stereo vision system for 3D imaging and reconstruction of greenhouse plants. Gan et al.^11^ employed structural similarity (SSIM) index to provide pixel-level registration for citrus canopy images. Kenta et al.^12^ collected plant images from 50 perspectives according to the SfM principle to realize the registration and 3D reconstruction of the plants. Colaco et al.^13^ utilized K-means and Alpha-shape algorithm to register and reconstruct citrus. Fadili et al.^14^ introduced a RegisTree algorithm to improve the quality of spatial registration of forests. The above algorithms have their characteristics, but the most widely used registration method is ICP algorithm. Vazquez-Arellano et al.^15^ considered ICP algorithm to reconstruct the 3D model of maize plants, and its reconstruction effect is pleasing, but the amount of calculation is too large during registration. Sun et al.^16^ chose ICP algorithm for the precise registration and reconstruction of multi-spectral 3D point cloud model of plants. However, the direct use of the ICP algorithm for point cloud registration will increase the amount of calculation and make the ICP algorithm become a local optimization, and eventually lead to the registration error being too large to meet the accuracy requirements^17^. There is still a lack of target-based improvement methods at present.

The purpose of this paper is to achieve the high-precision 3D reconstruction of maize plants by proposing an improved ICP-based algorithm, and the reconstructed model can be used to replace the maize plant for research to save resources and enhance the research efficiency. The novel aspects of this paper are summarized as follows:

1. A fully automatic point cloud collection platform was built to cooperate with a consumer-grade depth camera Kinect V2 to collect multi-angle point clouds of maize plants.
2. The conical surface fitting algorithm was put forward to perform coarse registration, the fitted rotation axis of point clouds was obtained by this algorithm and used to provide a better raw point cloud location for precise registration.
3. The interval ICP registration algorithm was proposed to perform precise registration, the method of interval registration was used for registration between the target point cloud and the point cloud to be registered, which can reduce the accumulation of registration error.

The rest of this paper is organized as follows. “Data collection” section presents the establishment of the collection system and the collection process of point clouds. The theoretical basis of coarse registration and precise registration of point clouds are discussed in “Registration process”, “Coarse registration based on conical surface fitting algorithm”, and “Precise registration based on interval ICP algorithm” section. Then experiments are set up to evaluate the performance of the collection platform and algorithm for reconstructing maize plants in “Result and discussion” section. Conclusion and future studies are summarized in “Conclusion” section.

## Materials and methods

The maize plant (*Zea mays* L. cv ‘Zhengdan958’) used in this study were obtained from the Experimental Field of College of Mechanical and Electronic Engineering, Shandong Agricultural University. The experiment was carried out at the MIE Research Center, College of Mechanical and Electronic Engineering, Shandong Agricultural University. The point cloud data of maize plants was collected through the self-built platform, and the conical surface fitting—ICP-based algorithm was used for point cloud registration. The specific methods are detailed in the following sections.

This study complies with the IUCN Policy Statement on Research Involving Species at Risk of Extinction and the Convention on the Trade in Endangered Species of Wild Fauna and Flora. All aspects of this study were conducted in compliance with relevant institutional, national, and international guidelines and legislation.

### Data collection

#### The establishment of the automatic point cloud collection platform

**Figure 1:**
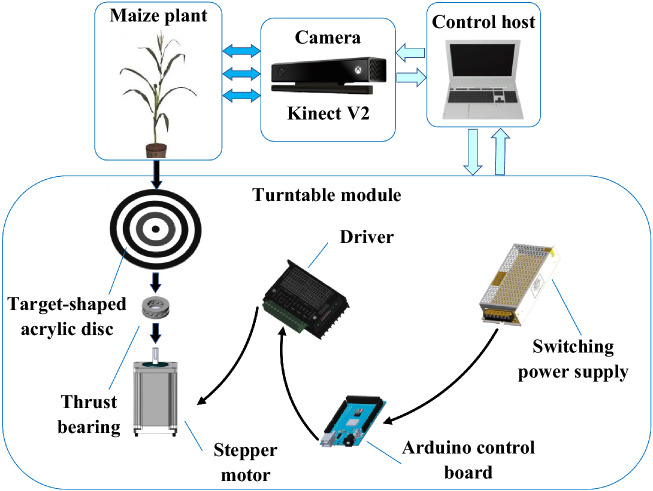
Schematic diagram of data collection platform.

In order to obtain ideal point clouds of maize plants, an automatic data collection platform was built, as shown in Fig. 1. The platform consists of three main modules: control host, turntable module, and camera module. A consumer-grade depth camera Kinect V2 was selected to collect color images and depth information of the maize plants. A high-precision turntable module was designed to realize the automatic rotation of the experimental objects. In the collection phase, under the control of the host, the turntable module and the camera module worked together to collect the multi-angle point clouds of maize plants.

The turntable module was composed of a target-shaped acrylic disc, a thrust bearing, an Arduino control board, a stepper motor, and a driver. In order to ensure that the axis of the flowerpot coincides with the rotation axis of the turntable during the data collection, the turntables’ tray was designed as a target-shaped disc. Meanwhile, the thrust bearing was used to support the disc on the stepper motor to provide sufficient supporting force, reducing the rotation resistance and ensuring the turntable module’s rotation accuracy.

#### Experimental object and environment setup

The effective viewing distance of the Kinect V2 is 0.5–4.5 m; and the maximum field of view angle is: 70° in the horizontal direction and 60° in the vertical direction, respectively^18,19^. Considering the limitation of the recognition range of the collection equipment and the width of the collection field of view, it is necessary to control the maize plant and the Kinect V2 within a proper distance. The maize plant at the flowering and kernel stage with shorter plant heights was selected as the experimental object, after the maize plant was transplanted into the flowerpot, the plant height above the upper face of the soil was about 1.2 m, and the diameter of the largest cylinder of the plant was 1.5 m.

The method of multi-angle point clouds fusion was adopted to complete reconstruction in this paper, which has high requirements for the morphological similarity between the collected datasets, environmental factors such as wind, natural light, and inter-plant contact under outdoor conditions have a significant impact on the accuracy of the collected data^20,21^. Then, the collection process was conducted in an experimental environment with an illuminance of about 170 lx, and the doors and windows were kept closed.

**Figure 2:**
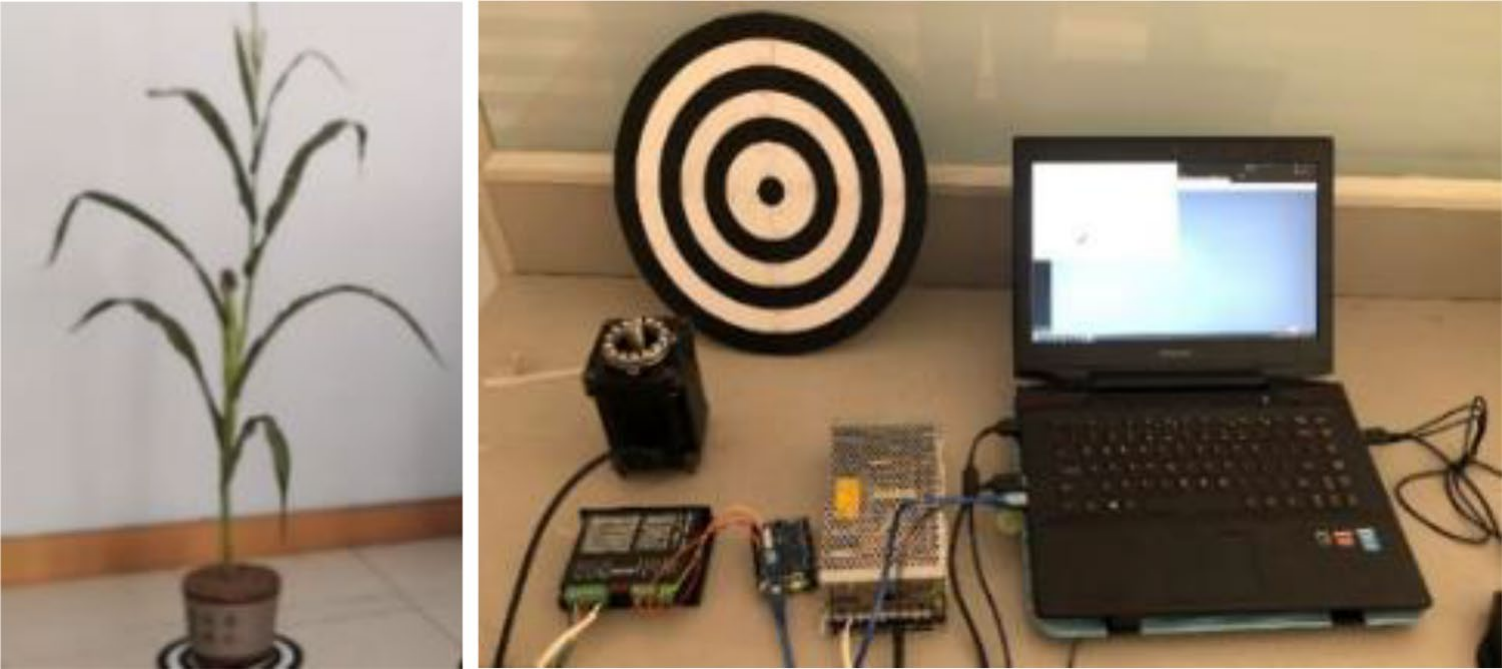
Experimental object and environment.

The experimental object and data collection platform are shown in Fig. 2.

#### Point clouds collection and preprocessing

**Figure 3:**
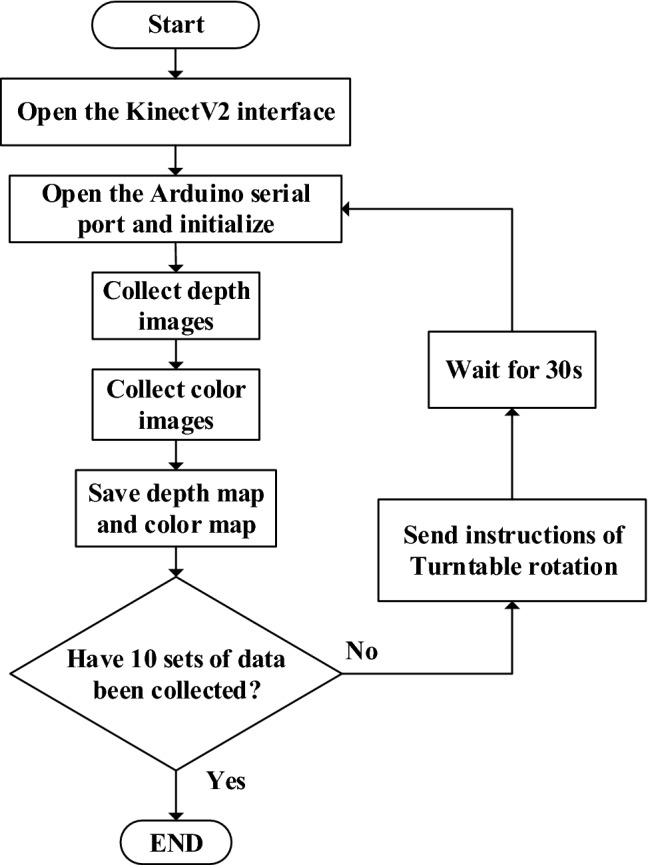
Flow chart of data collection process.

Nine times of 36° counterclockwise rotation and ten times of point clouds collection were performed in the data collection process. The collected point clouds were labelled as Plant_01–Plant_10. In order to avoid errors caused by elastic deformation of the maize plants during the rotations, a 30 s delay between each data collection process was performed. The data collection procedure is shown in Fig. 3.

The collected raw point clouds contain environmental point clouds and various noise, which would reduce the speed of data processing and the accuracy of 3D reconstruction. Then, the Pass-through filter algorithm was used to remove the environmental point clouds; and then the bilateral filtering algorithm was employed to smooth and denoise the rest point clouds^22–24^.

### Registration process

**Figure 4:**
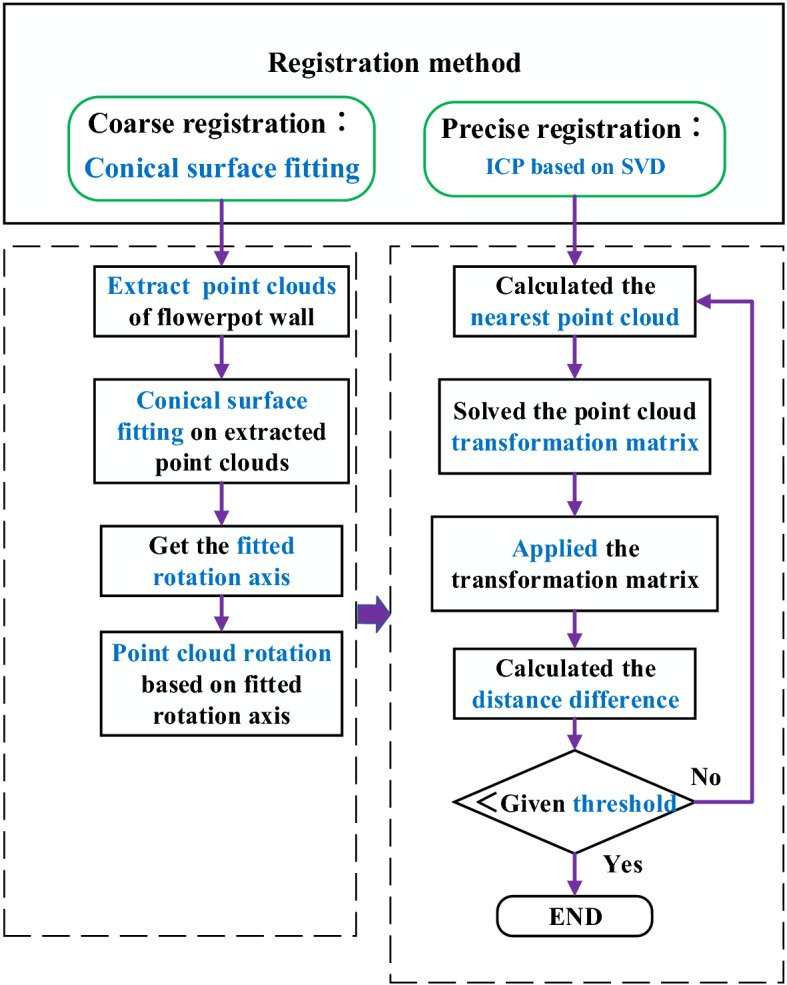
Flow chart of registration process.

The registration process is shown in Fig. 4, it includes a coarse registration and a precise registration. Since point clouds from different angles were collected by rotating maize plants, there is a problem that the coordinates of corresponding points on the point clouds from different angles were different obviously, the registration accuracy was easy to be unsatisfactory if the ICP was used for registration directly^25,26^. Therefore, the coarse registration based on the conical surface fitting algorithm was introduced, the algorithm was used to obtain the fitted rotation axis of point clouds in coarse registration by fitting the local point clouds of the flowerpot walls, thereby reducing the distance between corresponding points on the point clouds from different angles. Then, the interval ICP algorithm was employed to perform the precise registration, and Singular Value Decomposition (SVD) method was selected to complete the solution of the ICP^27,28^.

### Coarse registration based on conical surface fitting algorithm

If the ICP algorithm was used for point cloud registration directly, it would increase the calculation amount and make the ICP algorithm become a local optimization. The designed turntable module has ensured that the bottom center of the flowerpot would coincide with the rotor axis of the stepper motor, hence the central axis of the flowerpot wall was selected as the reference for coarse registration, the vector data of the central axis can be obtained by fitting, and then the fitted reference axis was used to complete the coarse registration of multi-angle point clouds for the subsequent precise registration.

#### Conical surface fitting on local point clouds of flowerpot wall

Considering the unguaranteed spatial posture of the flowerpot and the sufficient amount of point cloud data, the least squares method was selected to establish a conical surface fitting algorithm. The method to determine the fitted cone is to minimize the distance from the sum of all points in the point clouds to the fitting conical surface by adjusting the parameters:

1 $$mind(s) = \sum\limits_{{{\varvec{i}} = 1}}^{n} {d(s,{\varvec{P}}_{{\varvec{i}}} )^{2} } \, \left( {{\varvec{i}} = 1,2, \ldots ,n} \right),$$

where ***i*** are the points in the point clouds; *s* is the fitting parameter; *d(s)* is the residual sum of the point clouds of the fitting parameter *s*; d (s, ***P***_***i***_) is the distance between the point ***P***_***i***_ and the conical surface with fitting parameter *s*.

**Figure 5:**
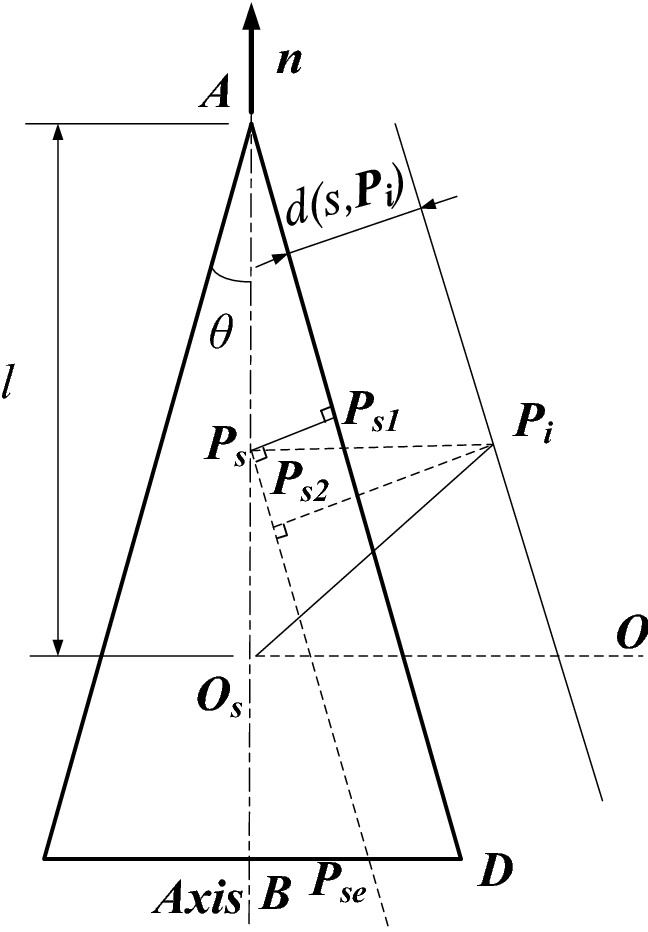
Distance function model of conical surface.

In order to obtain d (s, ***P***_***i***_), the distance function model of the conical surface was established. And the point clouds of flowerpot wall were assumed as the lower half cone model taken parallel to the bottom surface to reduce the parameter calculation, as shown in Fig. 5, where ***O*** is the coordinate origin, and ***O***_***s***_ is the projection position of the origin ***O*** on the cone axis. This figure takes a point ***P***_***i***_ in the point cloud as an example, ***P***_***s***_ is the projection of point ***P***_***i***_ on the cone axis*.*

The d (s, ***P***_***i***_) can be obtained by Eq. (2) after analyzing the distance relationships in Fig. 5, which can be equivalently expressed as Eq. (3):

2 $$d(s,{\varvec{P}}_{{\varvec{i}}} ){ = }\left| {{\varvec{P}}_{{\varvec{i}}} {\varvec{P}}_{{{\varvec{s2}}}} } \right| - \left| {{\varvec{P}}_{{\varvec{s}}} {\varvec{P}}_{{{\varvec{s1}}}} } \right| = \left| {{\varvec{P}}_{{\varvec{i}}} {\varvec{P}}_{{\varvec{s}}} } \right| \cdot \cos \theta - \left| {{\varvec{AP}}_{{\varvec{s}}} } \right| \cdot \sin \theta ,$$

3 $$d(s,{\varvec{P}}_{{\varvec{i}}} ) = \left( {{\varvec{O}}_{{\varvec{s}}} {\varvec{P}} - {\varvec{n}}\left\langle {{\varvec{O}}_{{\varvec{s}}} {\varvec{P}},{\varvec{n}}} \right\rangle } \right)\cos \theta - \left( {l - \left\langle {{\varvec{O}}_{{\varvec{s}}} {\varvec{P}},{\varvec{n}}} \right\rangle } \right)\sin \theta ,$$

where *θ* is the half cone apex angle; *l* is the length of the line segment between ***O***_***s***_ and the vertex ***A*** of the cone; ***n*** is the axial vector; ***P*** = ***P***_***i***_*** O***.

The distance function with constraints in the algorithm is:

4 $$d\left( {s,p} \right) = \lambda {\varvec{p}}^{2} - \left\langle {{\varvec{p}},\user2{n^{\prime}}} \right\rangle 2 + \left\langle {{\varvec{p}},\user2{s^{\prime}}} \right\rangle + A,$$

where $$k = \frac{1}{2l\sin \theta };\lambda = k(\cos \theta )^{2} ;\user2{n^{\prime}} = \sqrt k {\varvec{n}};\user2{s^{\prime}} = {\varvec{n}}\sin \theta - 2\lambda {\varvec{s}};A = \frac{{{\varvec{s}}^{2} - 1}}{4\lambda }$$.

The coordinate representation of vector data was introduced to facilitate calculation: ***p*** = (*x, y, z*), ***n*** = (*n*_*x*_*, n*_*y,*_* n*_*z*_), ***s′*** = (*s*_*x*_*′, s*_*y*_*′, s*_*z*_*′*), then set the parameter vector to ***S*** and point cloud data vector to ***P***:

5 $$\left\{ \begin{array}{*{20}l}{\varvec{S}} = (\lambda - n_{x}^{2} ,\lambda - n_{y}^{2} ,\lambda - n_{z}^{2} , - n_{x} n_{y} , \, - n_{x} n_{z} , - n_{y} n_{z} ,s_{x}^{\prime } ,s_{y}^{\prime } ,s_{z}^{\prime } ,A)^{T} \hfill \\ {\varvec{P}} = (x^{2} ,y^{2} ,z^{2} ,2xy,2xz,2yz,x,y,z,1)^{T} \hfill \\ \end{array} \right..$$

Then, ***S*** and ***P*** were substituted into the distance equation: *d (s, p)* = ***S ***^*T*^***P***, the least squares iterative objective function of ***S*** and ***P*** was obtained:

6 $$\min F(\user2{S,P}) = \sum\limits_{i = 1}^{n} {d(s,{\varvec{P}}_{{\varvec{i}}} )^{2} } = {\varvec{S}}^{T} \left( {\sum\limits_{i = 1}^{n} {{\varvec{P}}_{{\varvec{i}}} {\varvec{P}}_{{\varvec{i}}}^{{\varvec{T}}} } } \right){\varvec{S}}.$$

Then, the fitted rotation axes were different, in order to reduce the random error of the axial vector caused by this factor, Eq. (7) was used to calculate the mean vector of all the fitted rotation axis vectors as the rotation axis of the point clouds.

7 $${\mathbf{n = }}\frac{{\sum\nolimits_{j = 1}^{m} {{\mathbf{n}}_{j} } }}{m} \left( {j = 1,2, \ldots ,10} \right),$$

where *j* is the serial number of collections of the point clouds; ***n*** is the mean rotation axis vector; ***n***_***j***_ is the fitted rotation axis vector; *m* is the number of the fitted rotation axis vector.

#### Point cloud rotation based on the fitted rotation axis

The Plant_01 was used as the reference, the rest of the point clouds were rotated according to the collection sequence with a rotation angle of counterclockwise 36°. The matrix change during coarse registration was divided into three steps:

1. Translate the point clouds until the rotation axis passes through the coordinate origin ***O***.
2. Rotate the point clouds for the corresponding number of rotation times, which is the serial number of point clouds minus 1, respectively.
3. Reverse the translation opposite to step (1), translate the point clouds to restore the rotation axis to the raw position.

Among them, according to the 3D vector arbitrary axis (passing the origin ***O***) rotation Eq. (8), the rotation matrix of the point clouds could be calculated when the rotation axis passed the position of origin ***O***.

8 $${\varvec{R}}({\varvec{n}},\theta ) = \, \left[ {\begin{array}{*{20}l} {n_{x}^{2} (1 - \cos \theta ) + \cos \theta } & {n_{x} n_{y} (1 - \cos \theta ) + n_{z} \sin \theta } & {n_{x} n_{z} (1 - \cos \theta ) - n_{y} \sin \theta } \\ {n_{x} n_{y} (1 - \cos \theta ) - n_{z} \sin \theta } & {n_{y}^{2} (1 - \cos \theta ) + \cos \theta } & {n_{y} n_{z} (1 - \cos \theta ) + n_{x} \sin \theta } \\ {n_{x} n_{z} (1 - \cos \theta ) + n_{y} \sin \theta } & {n_{y} n_{z} (1 - \cos \theta ) - n_{x} \sin \theta } & {n_{z}^{2} (1 - \cos \theta ) + \cos \theta } \\ \end{array} } \right].$$

In summary, the transformation function of point clouds for coarse registration is expressed as:

9 $${\varvec{p}}_{j}^{\prime } = {\varvec{p}}_{j} \cdot {\varvec{T}}_{j} \cdot \left( {{\varvec{R}}({\varvec{n}},\theta )} \right)^{j - 1} \cdot {\varvec{T}}_{j}^{ - 1} \, \left( {j = 1,2, \ldots ,10} \right),$$

where ***p***_*j*_ is the point clouds with the serial number *n*; ***p***_*j*_*'* is the point clouds obtained by the matrix transformation of coarse registration of the ***p***_*j*_; *T*_*j*_ is the translation matrix.

### Precise registration based on interval ICP algorithm

#### Removal of point clouds of flowerpot

The percentage statistics of maize plant points were conducted before the ICP registration stage, as shown in Table 1. It can be seen that flowerpot accounts for 43.6–55.4% of the point clouds, which will reduce the registration efficiency of the maize plant and the accuracy of precise registration^29,30^. Therefore, the point clouds of flowerpot must be removed before the precise registration.

**Table 1:** Percentage of maize plant.

Serial number of point cloud	With flowerpot	Without flowerpot	Percentage of maize plant (%)
Plant_01	7803	3574	45.8
Plant_02	7345	3312	45.1
Plant_03	6952	3098	44.6
Plant_04	8424	4600	54.6
Plant_05	8640	4772	55.2
Plant_06	8159	4337	53.2
Plant_07	7463	3600	48.2
Plant_08	7586	3851	50.8
Plant_09	8840	4982	56.4
Plant_10	7876	4022	51.1

#### Interval ICP precise registration based on SVD

A number of registration algorithms have been proposed for 3D reconstruction tasks at present, such as ASW algorithm, K-means algorithm, Alpha-shape algorithm, and a RegisTree algorithm, but the most widely used algorithm is the ICP algorithm proposed by Besl and McKay^17^. The SVD can decompose any matrix in full order to compress the data to speed up the data processing, which was widely used in machine learning and image processing^31,32^. Therefore, SVD was selected to cooperate with the ICP for the precise registration process in this paper.

In the precise registration stage, the registration objective function is expressed as:

10 $$R^{*} ,t^* = arg_{R,t} min\frac{1}{{\left| {p_{s} } \right|}}\sum\limits_{i = 1}^{{\left| {P_{s} } \right|}} {\left\| {p_{t}^{i} - \left( {R \cdot p_{s}^{i} + t} \right)} \right\|}^{2} ,$$

where *p*_*s*_ are the corresponding points of the point clouds to be registered, *p*_*t*_ are the corresponding points of the target point clouds; *Ps* is the number of points of point clouds to be registered; *R, t* are the rotation matrix and the translation matrix of registration; R*, t* are the optimal solutions of *R* and *t* to be solved.

The objective model of the SVD in this paper is defined as:

11 $$\mathop {{{\min}}}\limits_{{\text{R,t}}} J = \sum \begin{gathered} n \hfill \\ i = 1 \hfill \\ \end{gathered} ||p_{i} - (Rp_{i}^{^{\prime}} + t)||_{2}^{2} ,$$

where *p*_*i*_ and *p*_*i*_′ are the point pair in the point clouds to be registered and the target point clouds; *p*_*i*_ – *(R p*_*i*_′ + *t)* is the error value of the *i*^th^ pair of points; *J* is the total error of the two-point clouds after registration. The model took the *J* as the dependent variable, the minimum of *J* can be solved by iteratively correcting the independent variable (*R* and *t*). Then R*, t* can be obtained.

The process of the ICP algorithm could be divided into four steps:

1. Determine the matching relationship between the point clouds to be registered and the target point clouds. The point-to-point method was used to retrieve the closest point between the point clouds to be registered and the target point clouds to determine the relationship of corresponding points in the two sets of point clouds^33^, which can ensure matching accuracy in this paper.
2. Use SVD to solve the least squares problem to obtain the transformation matrix (*R* and *t*). The transformation matrix was obtained to solve the optimal solution for ICP registration: the centroids (*p, p*′) of the point clouds to be registered and the target point clouds were calculated, and the de-centroid coordinates (*q*_*i*_, *q*_*i*_′) of each point were calculated.
  12 $$\left\{ \begin{gathered} q_{i} = p_{i} - p = p_{i} - \sum\limits_{i = 1}^{n} {p_{i} } \\ q_{i}^{\prime } = p_{i}^{\prime } - p^{\prime} = p_{i}^{\prime } - \sum\limits_{i = 1}^{{n^{\prime}}} {p_{i} }^{\prime } \\ \end{gathered} \right.$$
  The optimal solution R* was obtained by simplifying the objective model of Eq. (10), and t* was calculated according to the *R* obtained by Eq. (11).
  13 $$R^{*} = \arg \mathop {\min }\limits_{R} \frac{1}{2}\sum\nolimits_{i = 1}^{n} {||q_{i} } - Rq_{i}^{^{\prime}} ||^{2} ,$$
  14 $$t^{*} = p - Rp^{^{\prime}} .$$
3. Apply the transformation matrix to adjust the poses of the point clouds to be registered.
4. Calculate the distance between the transformed point clouds and the target point clouds. If it is less than the given threshold, stop the iterations and output the results, otherwise return to step (1).

**Figure 6:**
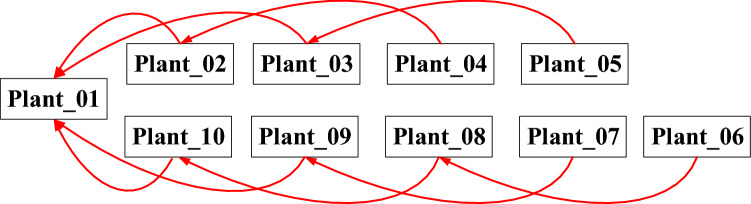
Schematic diagram of single-view point cloud registration target.

The interval registration method was selected to register ten sets of point clouds in this paper, as shown in Fig. 6.

## Result and discussion

### Results of coarse registration

**Figure 7:**
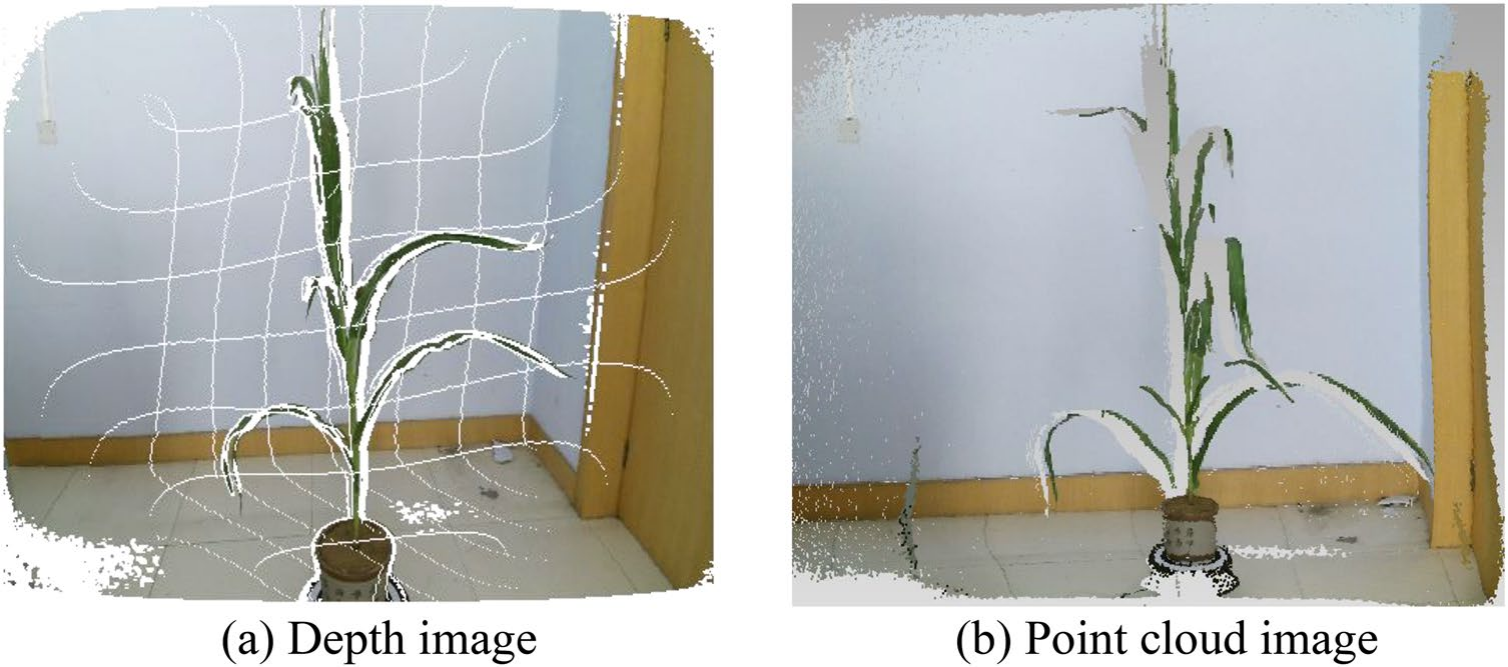
Depth map generates point cloud image.

**Figure 8:**
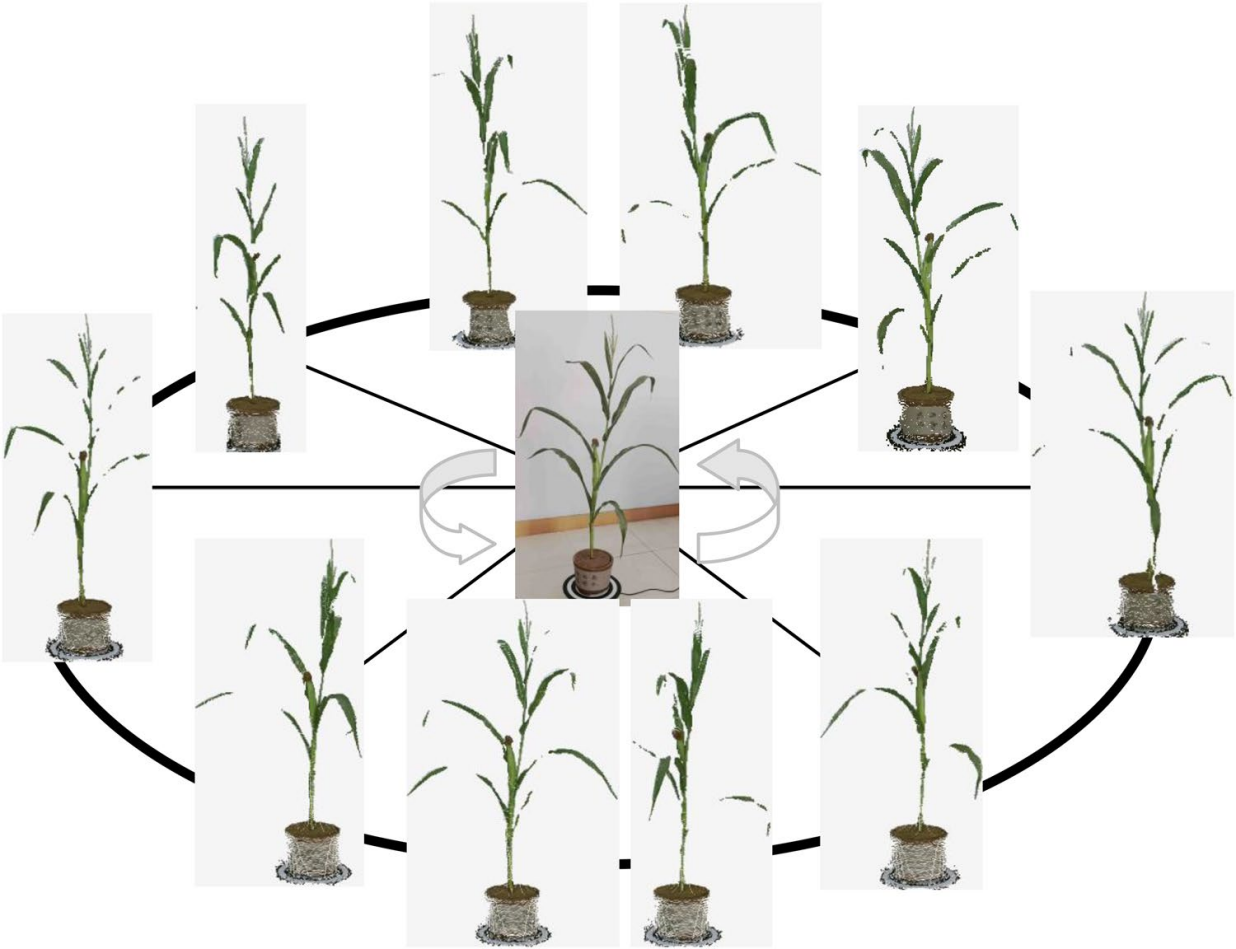
Point clouds from all angles.

The collected raw point clouds image and corresponding depth image of Plant_01 are shown in Fig. 7. Then, the ten sets of point clouds were performed environmental point cloud removal and denoising, as displayed in Fig. 8.

**Figure 9:**
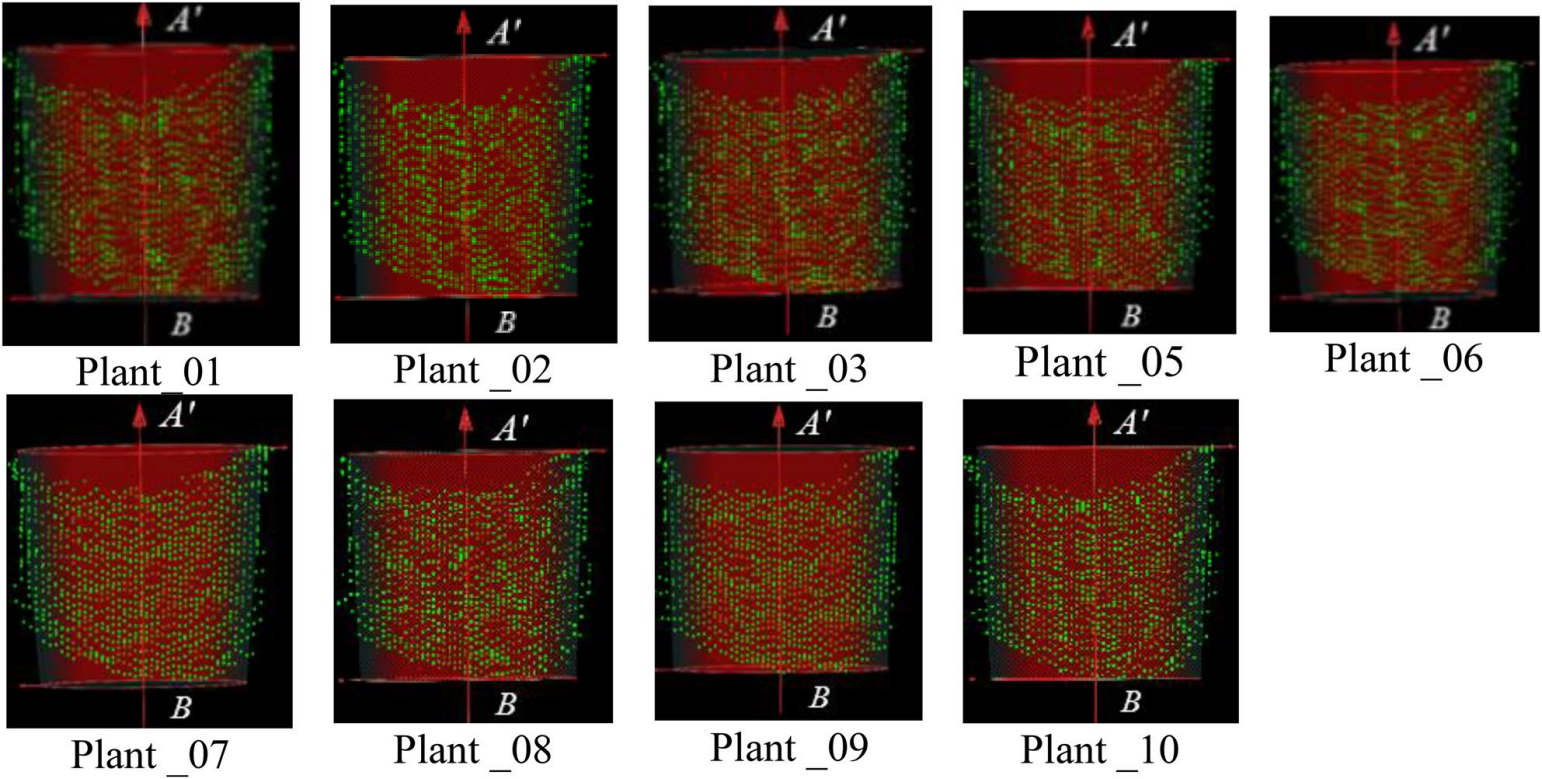
Effect diagrams of conical surface fitting of flowerpot wall.

Before conical surface fitting, the number of data points of point clouds on the flowerpot wall was counted, are shown in Table 2. It can be observed that the number of data points on the flowerpot wall of Plant_04 is relatively less than the other point clouds. To reduce the fitting error, the conical surface fitting was performed on the flowerpot walls of the other nine groups point clouds except for Plant_04. Since the inclination of the flowerpot wall was small, the truncated cone was intercepted at the positive farthest point of the rotation axis vector. Then the center of the upper bottom surface of the truncated cone was set as *A'*, the center of the lower bottom surface of the truncated cone was set as *B*. The fitting results are shown in Fig. 9.

**Table 2:** Statistics of flowerpot wall points.

Serial number of point cloud	Number of points
Plant_01	1053
Plant_02	1062
Plant_03	1065
Plant_04	966
Plant_05	1073
Plant_06	1073
Plant_07	1055
Plant_08	1066
Plant_09	1055
Plant_10	1075

The fitted rotation axes were solved after the conical surface fitting, the coordinates of *A'* and *B* on the fitted rotation axes and the rotation axis vectors are shown in Table 3.

**Table 3:** The coordinates of the top center point and the bottom center point, the fitted rotation axis vectors.

Serial number of point cloud	Top center (A′)/m	Bottom center (B)/m	Fitted rotation axis vector
Plant_01	(0.0262, − 0.5659, 1.6590)	(0.0247, − 0.7304, 1.7506)	(0.0015, 0.1645, − 0.0916)
Plant_02	(0.0267, − 0.5659, 1.6618)	(0.0240, − 0.7322, 1.7462)	(0.0027, 0.1663, − 0.0844)
Plant_03	(0.0282, − 0.5674, 1.6688)	(0.0231, − 0.7343, 1.7455)	(0.0051, 0.1669, − 0.0767)
Plant_05	(0.0238, − 0.5682, 1.6613)	(0.0224, − 0.7306, 1.7474)	(0.0014, 0.1624, − 0.0861)
Plant_06	(0.0246, − 0.5654, 1.6530)	(0.0212, − 0.7285, 1.7474)	(0.0034, 0.1631, − 0.0944)
Plant_07	(0.0251, − 0.5681, 1.6557)	(0.0223, − 0.7285, 1.7459)	(0.0028, 0.1604, − 0.0902)
Plant_08	(0.0257, − 0.5658, 1.6625)	(0.0230, − 0.7293, 1.7445)	(0.0027, 0.1635, − 0.082)
Plant_09	(0.0247, − 0.5693, 1.6654)	(0.0231, − 0.7336, 1.7441)	(0.0016, 0.1643, − 0.0787)
Plant_10	(0.0255, − 0.5665, 1.6592)	(0.0244, − 0.7329, 1.7469)	(0.0011, 0.1664, − 0.0877)

**Figure 10:**
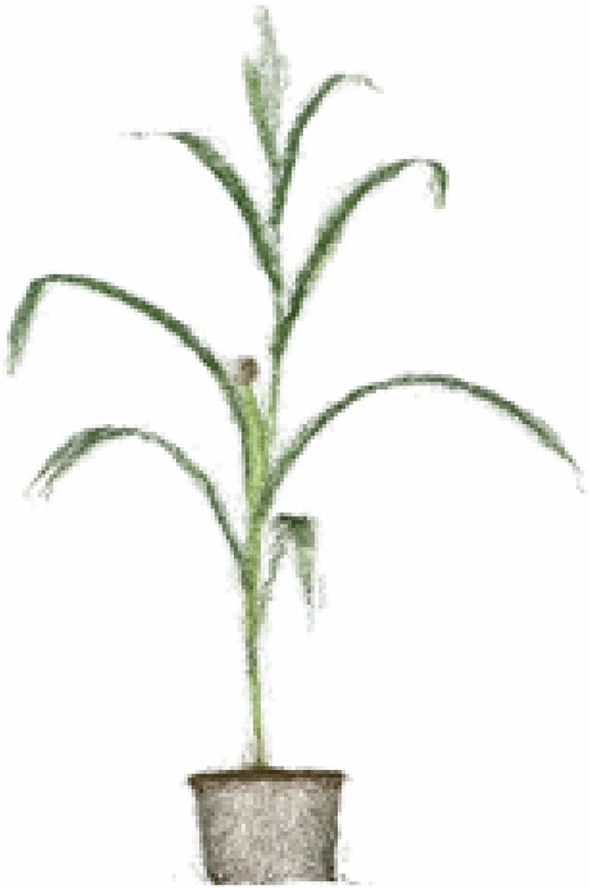
3D reconstruction model after coarse registration.

The coarse registration was performed on the point clouds of all angles based on the fitted rotation axis vectors. The results are displayed in Fig. 10.

### Results of precise registration

**Figure 11:**
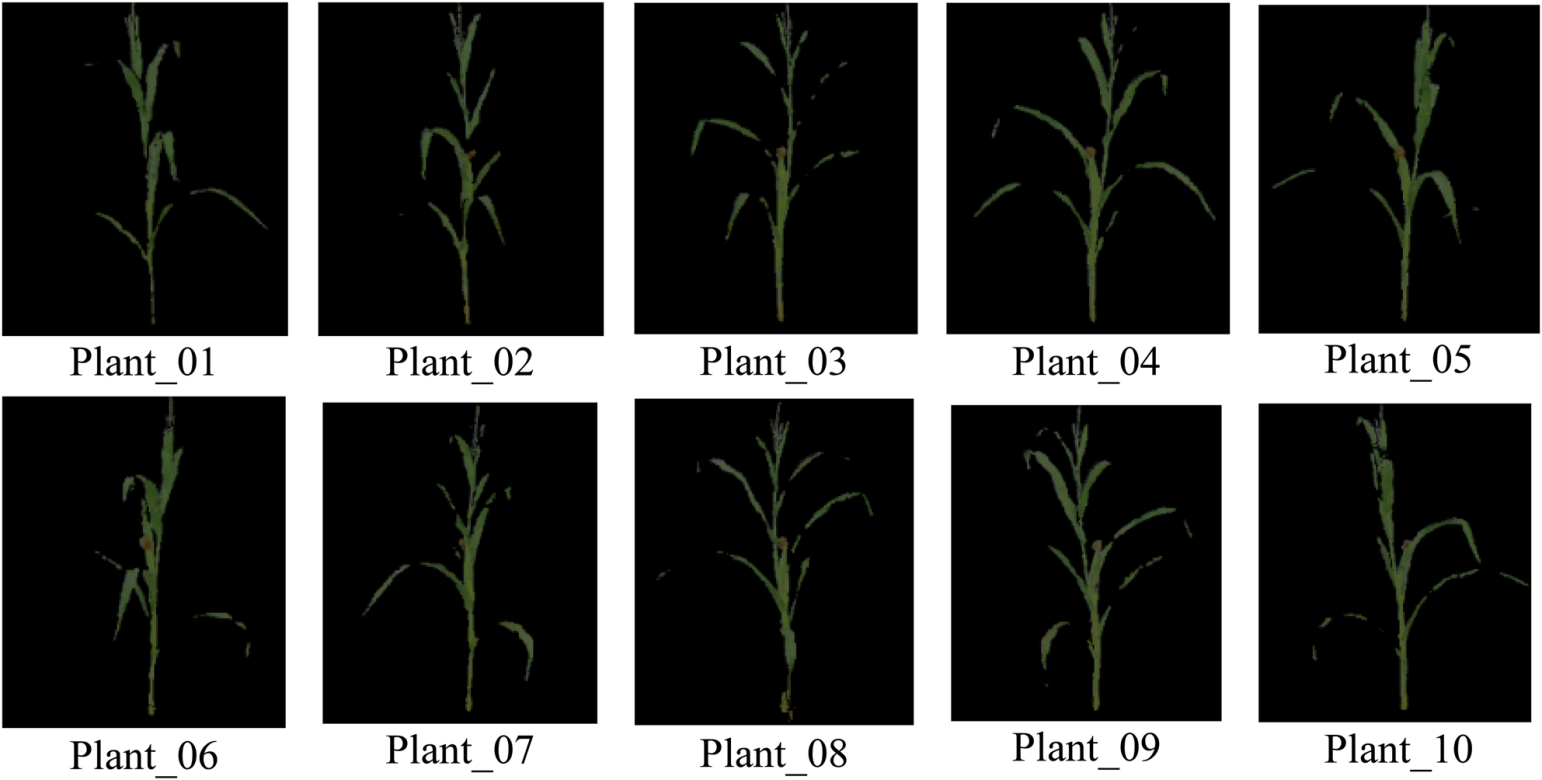
Remove the point clouds of flowerpot.

**Figure 12:**
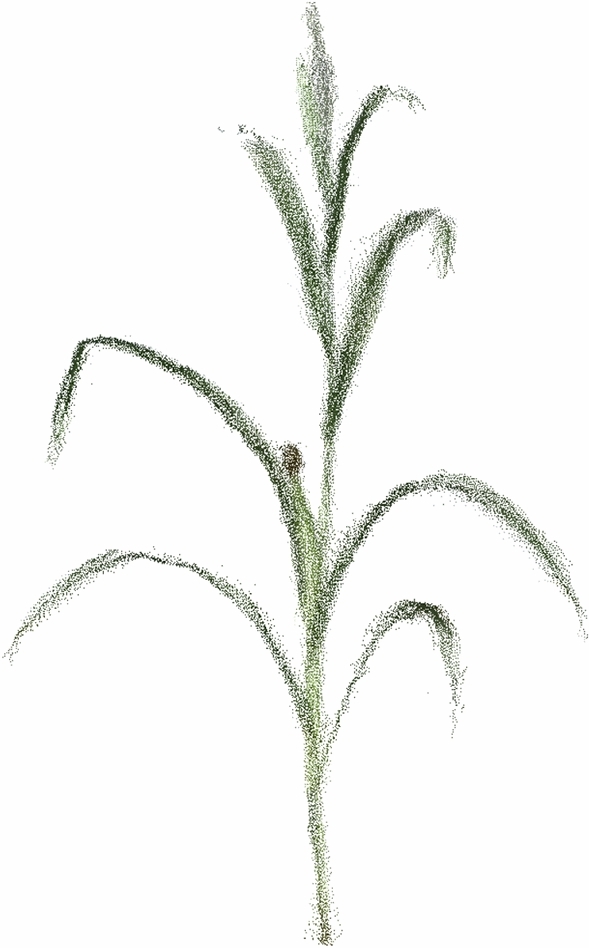
Effect diagrams of precise registration.

The point clouds of maize plants after removing the flowerpot are shown in Fig. 11. According to the interval registration sequence shown in Fig. 6, the ICP precise registration based on SVD was performed on the results of coarse registration. The number of registration iterations and the registration mean distance error (RMDE) between the corresponding points are shown in Table 4. The results of precise registration are shown in Fig. 12. According to statistical analysis, the RMDE of corresponding points of the precise registration under 10 angles is 1.98 mm, the comprehensive registration process took 57.32 s, which has high efficiency and registration accuracy.

**Table 4:** Statistics table of ICP registration result.

Point cloud to be registered	Target point cloud	Number of iterations	RMDE/mm
Plant_02	Plant_01	9	1.75
Plant_03	Plant_01	11	1.85
Plant_04	Plant_02	13	2.03
Plant_05	Plant_03	15	1.84
Plant_10	Plant_01	8	1.65
Plant_09	Plant_01	10	2.18
Plant_08	Plant_10	12	2.05
Plant_07	Plant_09	11	2.16
Plant_06	Plant_08	13	2.06

The registration algorithm proposed in this paper is an improved ICP-based algorithm. The interval registration method was used to reduce the accumulation of registration errors; the conical surface fitting algorithm was introduced to perform coarse registration to improve the registration efficiency. In terms of time complexity, the algorithm in this paper is ***O(n***^2^***)*** as the traditional ICP, where ***n*** is the size of the point set.

**Figure 13:**
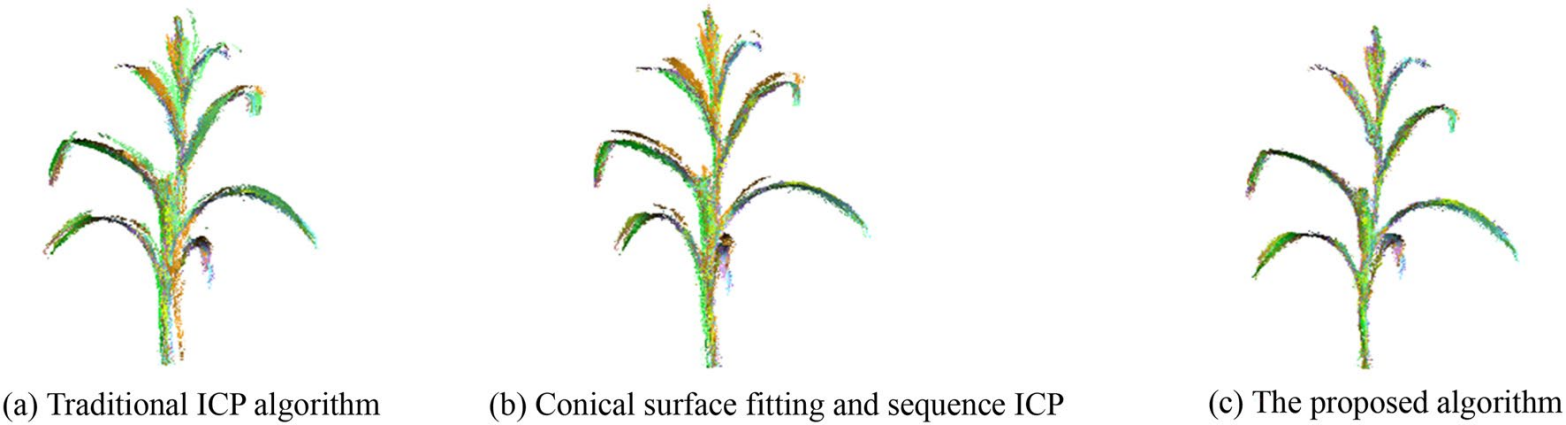
Comparison of registration results of three methods.

To verify the registration performance, the proposed algorithm was compared with the traditional ICP registration algorithm^17^ and conical surface fitting with sequential registration. The time consumption, mean number of iterations, and the RMDE of the three registration algorithms are listed in Table 5. The registration results are shown in Fig. 13.

**Table 5:** Comparison of ICP registration algorithms.

Method	Registration time/s	Mean number of iterations/times	RMDE/mm
Traditional ICP	102.36	23.88	10.65
Conical surface fitting with sequential registration	51.44	10.22	5.58
Conical surface fitting with interval registration (proposed in this paper)	57.32	11.33	1.98

Through the comparison and analysis of the results, it can be found:

1. Compared with the traditional ICP algorithm, coarse registration based on the conical surface fitting algorithm provides a better raw position for the precise registration. Observing the registration result of the traditional ICP algorithm in Fig. 13, it can be seen that there are individual point clouds which fall into the local optimal solution during the ICP registration. Because the distance between the corresponding points of the raw point cloud is relatively far, then more iterations of registration are required to converge the RMDE to the ideal state.
2. Compared with the interval registration method, the method of sequential point cloud registration can make the point clouds to be registered and the target point clouds have a better raw position, so there is a higher efficiency in the registration time and the number of iterations, but there is a phenomenon that the error value increases cumulatively as the registration object changes continuously according to the serial number.
3. The registration time of the proposed algorithm is shortened by 44.0% compared with the traditional ICP. Although the registration time is increased by 11.43% compared with the sequential registration method, the RMDE of the registration reduces by 81.41% and 64.51% respectively compared with the traditional ICP. The registration accuracy is improved significantly.

To verify the performance of the proposed algorithm, the recently published research on 3D reconstruction were compared, as shown in Table 6. In order to increase the credibility of the comparison, the algorithms participating in the comparison are ICP-based algorithms, and the experimental objects are agricultural or forestry crops. And the RMDE was selected as the performance indicates. It can be found that the RMDE of the proposed algorithm is about 2–5.6 mm less than other algorithms, which proves its superiority.

**Table 6:** Comparison of registration algorithms.

Source	Registration algorithm	Experiment object	RMDE/mm
This paper	Conical surface fitting registration—ICP	Maize plant	1.98
Reference^16^	Kinect sensor position calibration—ICP	Tomato plant	4.10
Reference^23^	Kinect sensor pose estimation and self-calibration—ICP	Tomato plant	4.60
Reference^24^	Random sample consensus—ICP	Lettuce	6.50
Reference^25^	Manual marking method—ICP	Jujube tree	7.60
Reference^26^	Improved SIFT—ICP	Green plant	4.80

### Evaluation of triangular meshing of registration results

In order to make the models of maize plants have 3D surface properties, the point clouds of maize plants were encapsulated in a triangular mesh. The Delaunay triangular meshing algorithm based on the Bowyer–Watson method was used to triangulate the point clouds of maize plants to complete the 3D reconstruction. The results are shown as follows.

**Figure 14:**
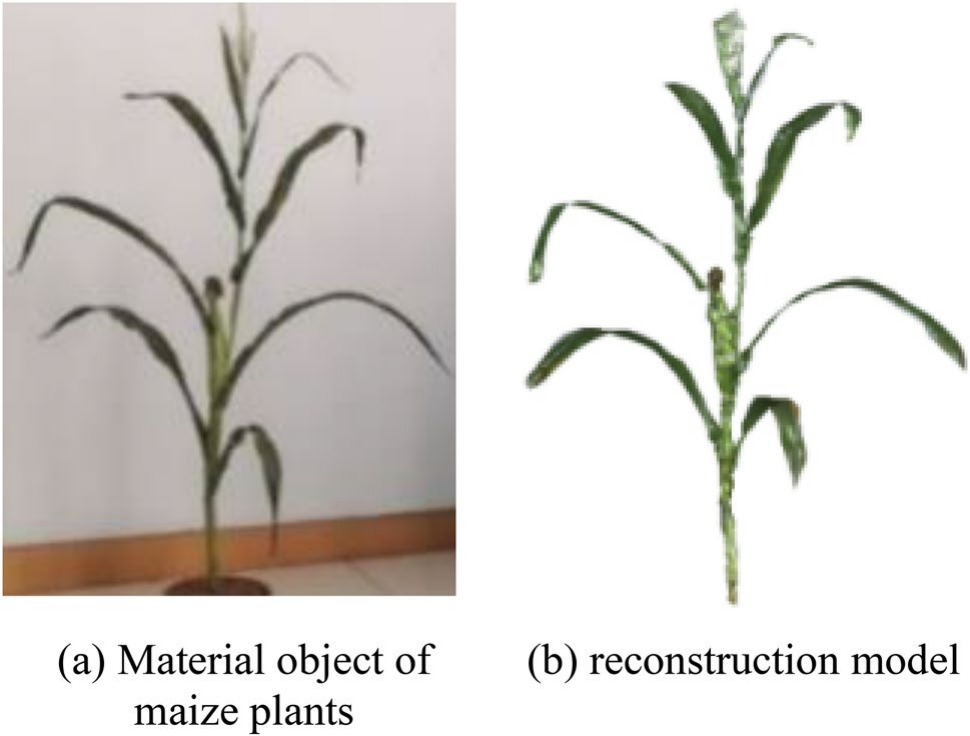
3D reconstruction results of maize plants.

**Figure 15:**
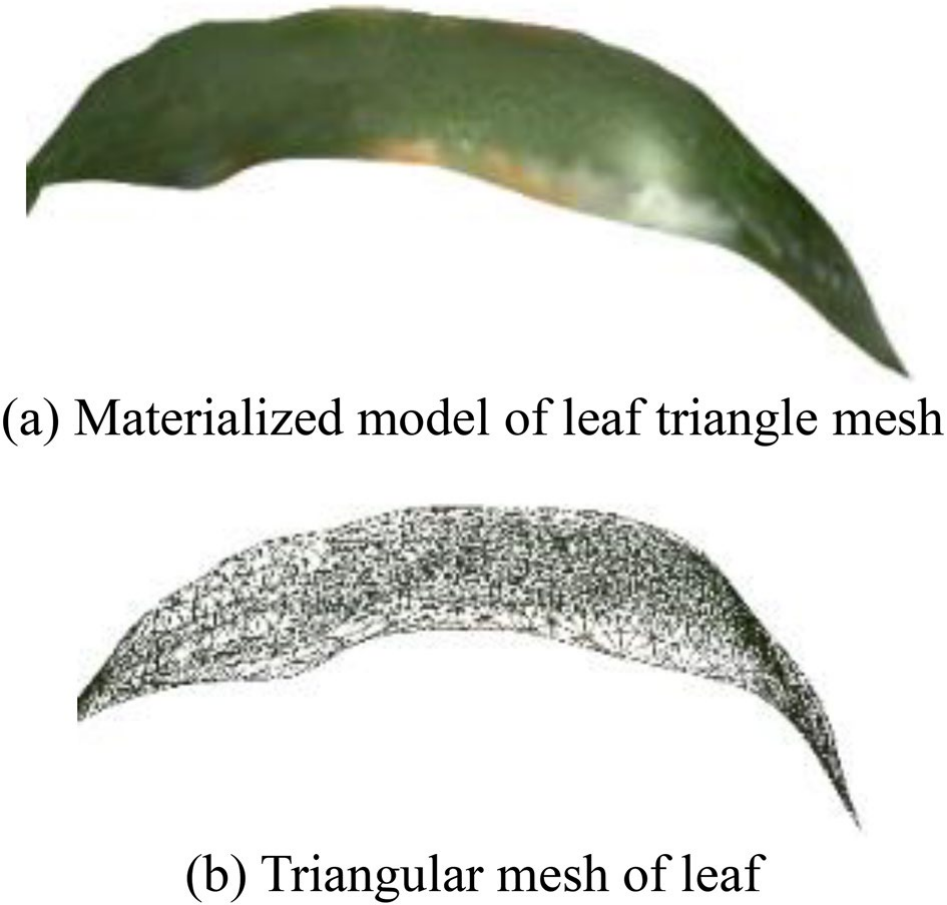
Triangular mesh results of leaf.

**Figure 16:**
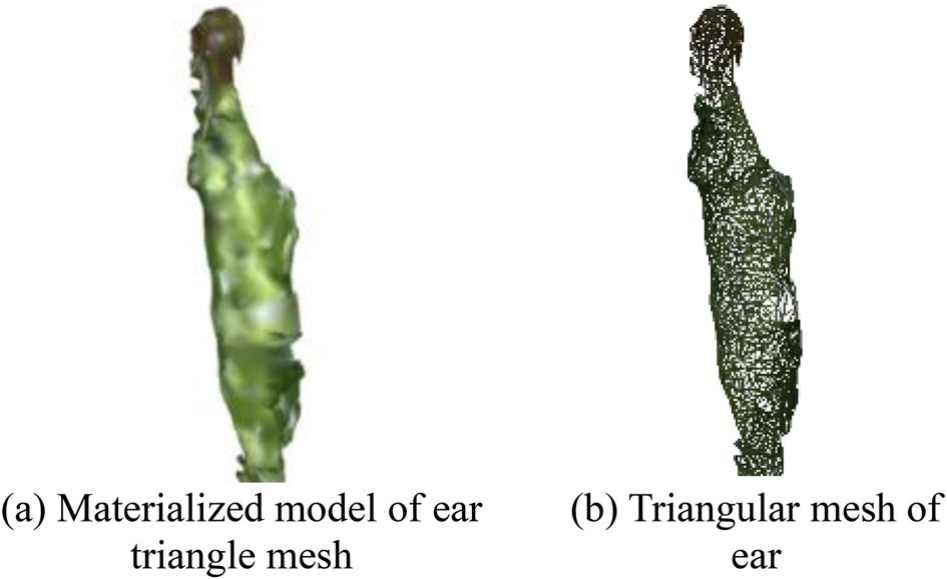
Triangular mesh results of ear.

Fig. 14 shows the raw image and the final 3D reconstruction model of the maize plants, respectively. It can be observed that the 3D reconstruction model has a high structural similarity. Fig. 15 displays materialized model of leaf triangle mesh and triangular mesh of leaf, it can be perceived that the materialized model has a relatively smooth curved surface, a non-closed structure and a higher degree of realism. Fig. 16 displays materialized model of ear triangle mesh and triangular mesh of ear, due to the close distance between the ear and the neighboring parts, there is a mesh packaging error caused by point cloud adhesion, but the overall structure similarity is high.

In order to analyze and verify the accuracy of the 3D reconstruction model of maize plants, the length of the leaf midline, the height of the plant above the upper face of the soil, and the perimeter at the maximum radius of the fruit were measured from the maize plant sample and 3D reconstruction model, as shown in Table 7. It can be found that the relative errors are controlled within 5%, and the maximum relative error is 4.90%. The model has a relatively reasonable proportional structure.

**Table 7:** Comparison of the measured values of key parts between sample and3D reconstruction model.

Object	Maize plant sample measured value/m	3D reconstruction model value/m	Error value (%)
Plant height	1.346	1.291	− 4.26
Maximum perimeter of ear	0.144	0.151	4.64
Leaf 1	0.657	0.627	− 4.78
Leaf 2	1.033	0.986	− 4.77
Leaf 3	0.938	0.907	− 3.42
Leaf 4	0.877	0.836	− 4.90
Leaf 5	0.644	0.617	− 4.38
Leaf 6	0.486	0.468	− 3.85

## Conclusion

In this paper, a point cloud registration method for maize plants based on conical surface fitting—ICP with an automatic point cloud collection platform was proposed. The key findings of this research and their significance are summarized below:

1. A consumer-grade depth camera Kinect V2 was employed with a point cloud collection platform to complete the automatic collection of point clouds.
2. The fitted rotation axis was used to replace the rotation axis of the point clouds for the coarse registration; the ICP interval registration algorithm based on SVD was employed to complete the precise registration.
3. The experimental results show that the coarse registration provides ideal raw point clouds for the ICP precise registration, the ICP algorithm using interval registration effectively reduces the error’s accumulation in the registration of the 10-angle point clouds. The full-angle registration takes 57.32 s, and the registration mean distance error is 1.98 mm, which has high registration efficiency and accuracy. The measured value’s relative errors between the maize plant sample and 3D reconstruction model are controlled within 5%, and the model has a relatively reasonable proportional structure.

Future research would be focusing on exploring more efficient algorithms to improve data processing efficiency under the condition of ensuring the quality of point cloud data processing, etc.

## Data Availability

The raw and processed data required to reproduce these results are available by contacting the author.
